# Embryonic Exposure to Tryptophan Yields Bullying Victimization via Reprogramming the Microbiota-Gut-Brain Axis in a Chicken Model

**DOI:** 10.3390/nu14030661

**Published:** 2022-02-04

**Authors:** Xiaohong Huang, Jiaying Hu, Haining Peng, Heng-wei Cheng

**Affiliations:** 1 Institute of Neuroregeneration & Neurorehabilitation, Department of Pathophysiology, Qingdao University, Qingdao 266071, China; 2 Department of Animal Sciences, Purdue University, West Lafayette, IN 47907, USA; hu165@purdue.edu (J.H.); hwcheng@purdue.edu (H.-w.C.); 3 Department of Sports Medicine, The Affiliated Hospital of Qingdao University, Qingdao 266071, China; haining_peng@hotmail.com; 4 Livestock Behavior Research Unit, USDA-ARS, West Lafayette, IN 47907, USA

**Keywords:** tryptophan, catecholamines, gestational stress, microbiota composition, gut-brain axis, aggression, bullying

## Abstract

Maternal metabolic disorder during early pregnancy may give rise to emotional and behavioral disorders in the child, vulnerable to bullying. Placental tryptophan fluctuation consequently disrupts offspring gut microbiome and brain neurogenesis with long-lasting physiological and social behavioral impacts. The aim of this study was to examine the hypothesis that the excess gestational tryptophan may affect children’s mental and physical development via modifying the microbiota-gut-brain axis, which lays the foundation of their mental status. Chicken embryo was employed due to its robust microbiota and independence of maternal influences during embryogenesis. The results indicated that embryonic tryptophan exposure reduced body weight and aggressiveness in the male offspring before and during adolescence. Additionally, the relative gut length and crypt depth were increased, while the villus/crypt ratio was decreased in tryptophan treated roosters, which was corresponding to the changes in the cecal microbiota composition. Furthermore, the catecholamine concentrations were increased in tryptophan group, which may be associated with the alterations in the gut microbiome and the gut-brain axis’s function. These changes may underlie the sociometric status of bullying; clarify how gestational tryptophan fluctuation compromises bullying and provide a strategy to prevent bullying by controlling dietary tryptophan and medication therapy during pregnancy.

## 1. Introduction

Bullying during childhood is linked to social anxiety, as a major public health issue, which often lasts into adulthood and increases the risk of personality disorders, causing life-long psychological damage to both bullies and bullying victims. To date, genetic, gestational, and environmental effects on these social behavioral roles have been reported [1]. Sociological studies have debated and noted that some children can be bullies by nature rather than learning [2]. Infants’ physicality, showing their bodily force to grab others’ toys, is a normal but emotionally arousing situation [2]. Babies born to stressed mothers are easier prey for bullies, i.e., the experiences during embryogenesis may reprogram the fetal development, increasing vulnerability to be bullied postnatally [3], which is associated with the fetal origins of adult disease hypothesis [4]. However, it is not well known how the maternal risk factors translate during embryogenesis with long-lasting effects on social relationships in later life.

Tryptophan (Trp), as an essential amino acid, is mainly derived from the diet in the small intestine and, as the sole precursor of serotonin (5-HT), is directly and indirectly involved in modulating brain function [5], which plays a critical role in developing social-emotional behaviors in offspring [6]. The altered Trp metabolism is one of the core biological signatures of prenatal maternal stress [7] caused by intrauterine inflammation, increasing the potential risk of neurodevelopmental disorders (NDDs), such as autism spectrum disorders (ASD) [8], which is closely related to bullying in youth [9,10,11]. Urinary Trp and purine metabolites are consistently upregulated in the gestational diabetes mellitus patients [12], and excessive Trp compromises pregnancy, e.g., pre-eclampsia [13]. In ASD children, the impaired Trp metabolism has been detected in the central nervous system (CNS), blood, and gut, which may underpin the pathophysiology of ASD [14,15]. In addition, gut microbiota work as a virtual endocrine organ [16]. Prenatal stress disrupts placental Trp metabolism, altering Trp availability during fetal neurogenesis due to reductions in the maternal Trp-metabolizing microbes [17]. Concerning the connection between the gastrointestinal symptoms and NDDs [14,18], multihits early-life stress alters brain gene expression and social communication, laying the foundation of physical and mental health in adulthood, by reprogramming their gut microbiome [19,20]. Moreover, bullying situations have been proven in hamsters to reduce the gut microbiota diversity, by acutely and repeatedly exposing Syrian hamsters to social stress [21].

The interaction between maternal gut microbiome and Trp metabolism involves in NDDs in children [22]. The excess Trp supply during pregnancy may affect the sociometric status in bullying via modifying the microbiota-gut-brain (MGB) axis. However, NDDs are affected by multiple factors including environmental stimulations, being difficult to identify the underlying mechanisms. For example, the mother-to-child transmission of microbes during pregnancy, labor delivery, and breastfeeding is irresistible in viviparous animals [23], which causes inevitable background noise in studying the influences of gestational Trp fluctuation on the offspring microbiome. Avian species have been central to investigate the ontogenetic origins of behaviors due to the directly controllable embryogenesis for the oviparous animals [24], i.e., chickens are currently an elective model system for investigating ASD [25,26]. Moreover, within flocks, chickens have a social hierarchy referred to as a pecking order: peckers within a flock always start feather pecking at others, as a reason to avoid adding young birds or small birds to old flocks. Hence, peckers within a flock could be as a comparison of bullies within a social group [27]. Comparing vertebrate embryonic development among various animals indicates that human embryos initially share characteristics in common with avian embryos [28]. Chicken embryos have been validated and used in various human biological, pharmacological, and immunological studies [29]. Plenty of Trp from the yolk and albumin [29] is consistent with the high Trp availability in the mammal umbilical cords. Thus, chicken embryo was recruited in studying the effect of excessive embryonic Trp on postnatal bullying and related changes in the MGB axis due to its similar genetic background and robust microbial community [30] but independence of maternal influences on the development of neural and digestive systems. Trp administration was conducted at embryonic day (E) 12, the body weight (BW) and aggressiveness were tracked, and the intestinal microbiome and brain neurotransmission were determined during adolescence. The results of this study may shed light on how the gestational Trp exposure affects peer relationships in later life and hint at the perinatal or early postnatal interventions for preventing or reducing bullying among individuals, especially those who experienced prenatal maternal stress.

## 2. Materials and Methods

### 2.1. Eggs Incubation and Embryonic Trp Exposure

**Figure 1:**
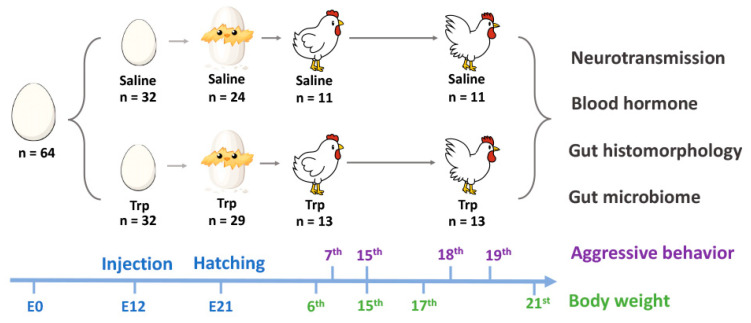
The graph of the experimental design.

A total of 64 fertilized eggs with similar weight were collected from the W-36 (Hy-Line Farms Inc., West Des Moines, IA, USA) White Leghorn hens within three days. The eggs were set in an incubator (NatureForm, Inc., Jacksonville, FL, USA) with a temperature at 99.9 °F and humidity of 60%. Egg candling was conducted at E7, E14, and E21 [31] and all the unhatched eggs were opened at the end of incubation to determine the rate of fertilization, embryonic mortality, and hatchability. As indicated in Figure 1, at E12, the embryos were randomly assigned into two groups (*n* = 32 per group): control group (100 uL saline per eggs) and Trp group (500 µg in 100 uL saline per egg). The Trp exposure was conducted by injecting the Trp solution or saline into the air sac followed the procedure reported previously [32]. The dosage of Trp and timing of injection was decided based on the outcomes of the pilot studies (Supplementary Table S1).

### 2.2. Bird Management

The hatch rate (i.e., the number of chicks born alive) was at approximately 83% without treatment effects. Fifty-three birds were hatched (Saline: 24; Trp: 29) and marked with wing bands at the hatchery, then the birds were transferred to the grower facility and kept separately based on treatment. The genders were determined at the end of the first week and the housing of the birds were rearranged based on gender (Saline: 11 males, 13 females; Trp: 13 males; 16 females). To avoid gender effects and physical bulling tends more often among boys [33], we focused on the effects of Trp exposure on the rooster development and associated aggressive behavior in this study. In the 16th week, all the birds were transferred to the layer facility and housed in single bird cages. The bird management, including the room temperature and humidity, lighting program, diet, and water, was provided according to the management guide of Hy-36 [34]. The protocol of this study was approved by the Purdue University Animal Care and Use Committee (Number: 1712001656).

### 2.3. Sample Collection

The BW of the birds were collected at week 6, 15, 17, and 21. At the 21st week, the roosters (n_Saline_ = 11, n_Trp_ = 13) were scarified by cervical dislocation, immediately following blood sampling. The blood samples were collected by cardiac puncture and placed into EDTA-coated test tubes, then centrifuged for 15 min at 700 g to collect plasma. The plasma samples were stored at −80 °C until the enzyme-linked immunosorbent assay (ELISA). The weight of the spleen, liver, and testes of each sampled bird were recorded immediately. The length of the entire small intestine and its sections, including the ileum, jejunum, and duodenum, were measured. Then the junction between the ileum and jejunum (approximately 1 cm) was collected and kept in 4% paraformaldehyde solution at room temperature for histomorphological analysis. The cecal content was collected using sterilized tools, snap frozen in liquid nitrogen, then stored at −80 °C for the microbiome gene sequencing. The brain was dissected based on the previously reported protocol [35], the raphe nuclei (RN) and HP including the thalamus and hypothalamus were collected and stored at −80 °C for high-performance liquid chromatography (HPLC) analyses.

### 2.4. Aggressive Behavior Observation

The aggressive behavior observation was conducted in a novel cage at the age of 7th, 15th and 18th weeks (Saline vs. Trp; n_7th_ = 7 pairs, n_15th_ = 6 pairs, n_18th_ = 9 pairs), and 19th week (Saline vs. Saline, Trp vs. Trp; n_19th_ = 4 pairs). The rationale and cellular mechanisms of the test is like the resident-intruder test which is a standardized method used in rodents for detecting social stress-induced aggression and violence [36]. Birds were paired based on their BW, and behaviors were recorded to a DVR system for a 30-min period. Frequency and duration of aggressive behaviors and feather pecking behaviors were identified and analyzed according to the previous study conducted in our lab [37]. The behavioral ethogram is in Table 1.

### 2.5. Intestinal Histomorphological Analysis

The intestinal samples were processed following the previous published protocol [38]. Briefly, the intestinal samples were dehydrated with ethanol, cleared with xylene, embedded with paraffin wax, and cross sectioned at a thickness of 5 μm. The paraffin sections were sequentially stained with hematoxylin and eosin following the instruction of the kit (GeneCopoeia, Rockville, MD, USA). Images were taken with Olympus BX40 F-3 microscope (Olympus Cooperation, Tokyo, Japan) attached to a digital video camera (Q-imaging 01- MBF-200R-CLR-12, Tucson, AZ, Canada). The software of ImageJ (National Institutes of Health, Bethesda, MD, USA) was used to determine the villus height and crypt depth by randomly measuring 10 villi from each sample. The mean was used for statistical analysis.

### 2.6. 16S rRNA Microbiome Sequencing

The total DNA was extracted from the frozen cecal contents using the QIAamp Fast DNA Stool Mini Kit (Qiagen, Hilden, NRW, Germany). Total DNA concentration of each sample was determined using NanoDrop (ND-1000 Spectrophotometer, ThermoScientific, Wilmington, DE, USA). 16S rRNA amplicon sequencing was performed using Illumina paired-end sequencing by the Environmental Sample Preparation and Sequencing Facility (ESPSF) of Argonne National Laboratory (Chicago, IL, USA). The V4 region of the 16S rRNA gene (515F-806R) was PCR amplified with region-specific primers that included sequencer adapter sequences used in the Illumina flowcell [39,40]. Briefly, sample DNA was amplified in a 25 μL PCR reaction system containing MO BIO PCR Water (Certified DNA-Free), 1x QuantaBio’s AccuStart II PCR ToughMix, 200 pM of each Golay barcode tagged Forward Primer and Reverse Primer, and 1 μL of DNA template. The temperature cycling conditions were as follows: 94 °C for 3 min, with 35 cycles at 94 °C for 45 s, 50 °C for 60 s, and 72 °C for 90 s; and then 10 min at 72 °C. Amplicons were quantified by PicoGreen (Invitrogen, ThermoScientific, Wilmington, DE, USA) and a plate reader (InfiniteÒ 200 PRO, Tecan, Männedorf, Switzerland), equally pooled into a single tube, cleaned up using AMPure XP Beads (Beckman Coulter, Pasadena, CA, USA), and then quantified by a fluorometer (Qubit, Invitrogen). Afterwards, the molarity of the pool is determined and diluted down to 2 nM, denatured, and then diluted to a final concentration of 6.75 pM with a 10% PhiX spike for sequencing on the Illumina MiSeq. Finally, the amplicons were sequenced on a 151 bp × 12 bp × 151 bp MiSeq run using customized sequencing primers [39]. The sequencing data were in FASTQ format.

### 2.7. ELISA

Plasma concentrations of Trp were detected in duplicate using the commercial ELISA kit (MBS038462, MyBioSource, San Diego, CA, USA). The optical density of each sample was read at 450 nm (Epoch Microplate Spectrophotometer, BioTek, Winooski, VT, USA). All samples were measured in duplicates with coefficient of variability (CV) ≤ 15%.

### 2.8. HPLC

The neurotransmitter concentrations in the brain tissue were detected using HPLC analyses following the previous description [37]. The concentrations of 5-HT, 5-hydroxyindoleacetic acid (5-HIAA), Trp, dopamine (DA), epinephrine (EP), and norepinephrine (NE) were obtained through each relative standard reference curve, respectively, and then the 5-HT turnover rate (5-HIAA/5-HT ratio) was calculated.

### 2.9. Data Processing and Analysis

#### 2.9.1. Bioinformatics Analysis

Paired-end reads were preprocessed using Trimmomatic 0.35 software [41] and assembled using FLASH 1.2.11 software [42]. Assembling parameters were: 10 bp of minimal overlapping, 200 bp of maximum overlapping, and 20% of maximum mismatch rate. The sequences were performed further denoising using the split_libraries 1.8.0 [43] following the criteria: reads with ambiguous, homologous sequences or below 200 bp were abandoned, and reads with 75% of bases above Q20 were retained. Chimeric sequences were deleted by UCHIME 2.4.2, then obtaining valid tags with a higher quality sequence [44]. The valid tags with a distance-based similarity of 97% or higher were grouped into OTUs using Vsearch 2.4.2 software [45]. The representative tags of each OUT were annotated by the RDP classifier (confidence threshold = 70%) [46].

The taxonomy of species annotation was divided into six levels, including phylum (L2), class (L3), order (L4), family (L5), and genus (L6). The alpha diversity analyses, including the alpha diversity index (Simpson, Shannon, Goods coverage, etc.), estimated OTU richness (Chao1), and species accumulation curve, were conducted using Mothur 1.32.1 software to compare the mean diversity between the Trp and Saline groups [47]. Principal component analysis (PCA), the beta diversity analysis, based on the Bray–Curtis similarities of OTU composition, was applied to rank the bacterial communities. Analysis of similarities (ANOSIM) was used to analyze the difference between the Trp and Saline groups based on the abundance of OTUs [48].

Least discriminant analysis effect size [LEfSe, linear discriminant analysis (LDA) coupled with effect size measurements] were applied to distinguish the difference in the dominant bacterial communities. Significant differences between the microbial communities were analyzed using the Wilcoxon rank sum test. Differences were considered significant if the *p* ≤ 0.05.

Phylogenetic investigation of communities by reconstruction of unobserved states (PICRUSt) [49] was used to predict functional genes of the classified members of microbiota resulting from reference-based OTU picking against Greengenes database [50]. Predicted genes were then hierarchically clustered and categorized under Kyoto Encyclopedia of Genes and Genomes (KEGG) orthologs [51].

#### 2.9.2. Physiological and Behavioral Data

The relative organ weight was calculated as: (absolute organ weight (g)) ⁄ (BW (kg) × 100%), while the relative gut length was calculated as: Gut length (cm)/(BW (kg). Physiological data were subjected to one-way ANOVA using PROC MIXED of SAS 9.4 software (SAS Institute Inc., Cary, NC, USA) to determine the effect of Trp exposure, or Two-way ANOVA using PROC MIXED to determine the age and Trp exposure effects. Data transformation (Box-Cox transformation) was performed to improve normality [52]. Behavioral durations were analyzed using PROC MIXED (SAS 9.4) and frequencies were analyzed using PROC GLIMMIX (SAS 9.4). Data were present as least square means (LSMeans) ± standard error of the mean (SEM). Statistical difference was set at *p* ≤ 0.05, and a trend was reported at 0.05 < *p* ≤ 0.10.

## 3. Results

### 3.1. Embryonic Trp Exposure Reduced BW

**Figure 2:**
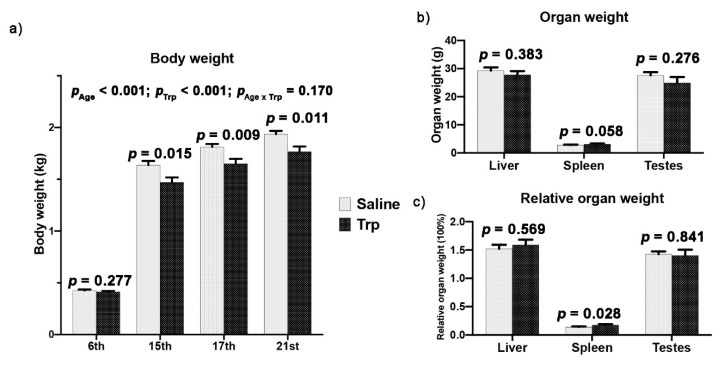
The effect of embryonic Trp exposure on BW and organ weight. (**a**) The body weight of birds at different age stages from the 6th to 21st week (n_Saline_ = 11; n_Trp_ = 13). (**b**–**c**) The absolute and relative weight of the liver, spleen and gonad at the 21st week (n_Saline_ = 11; n_Trp_ = 13).

The developmental and Trp exposure effects on the BW were revealed from the 6th to 21st week in male birds, before and after their sex maturity (Figure 2a, *p*_Age_ < 0.001, *p*_Trp_ < 0.001, *p*_Age x Trt_ = 0.170). Significant decrease in the BW by Trp exposure was revealed at the 15th, 17th and 21st weeks (Figure 2a). The absolute and relative weights of organs, including the liver, spleen, and testes, were not altered by Trp at the 21st week (Figure 2b,c) except that an increase in the relative spleen weight was found in Trp roosters (Figure 2c).

### 3.2. Embryonic Trp Exposure Reduced Aggressiveness

**Figure 3:**
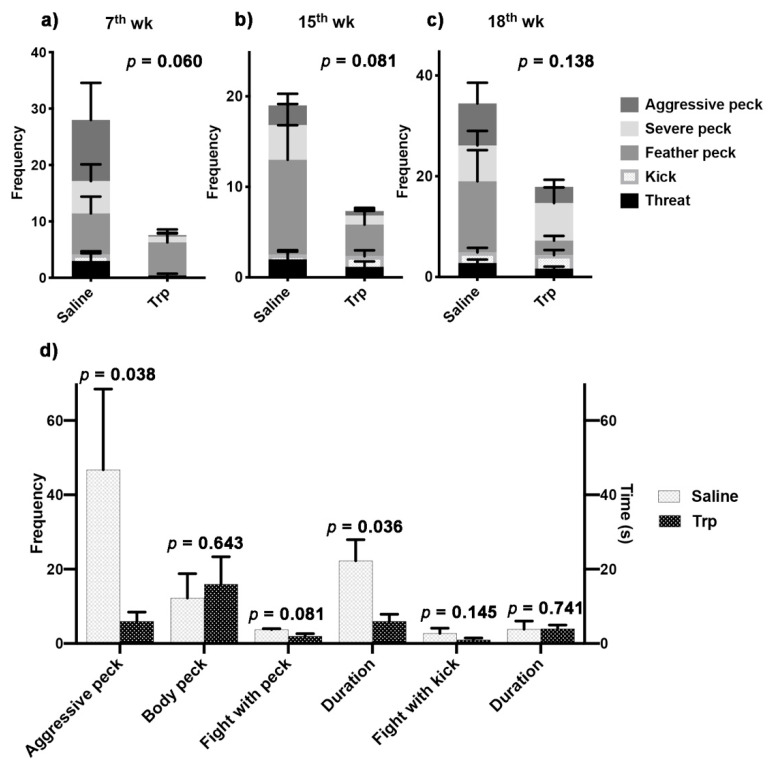
The effect of embryonic Trp exposure on aggressive behaviors. (**a–c**) The outcomes of the aggressive behavior tests between the Trp and Saline groups at the 7th, 15th and 18th weeks (Saline vs. Trp; n_7th_ = 7 pairs; n_15th_ = 6 pairs; n_18th_ = 9 pairs). The *p*-value indicated the significant level of the pooled frequency of all the behaviors between treatments. The statistical analysis of each behavior is attached in Table 2. (**d**) The outcomes of the aggressive behavior test within Saline or Trp group at the 19th week (Saline vs. Saline, Trp vs. Trp; n_19th_ = four pairs). The behavioral ethogram is in Table 1.

Aggressive behavioral tests were conducted between the Trp and Saline birds to determine the effect of Trp exposure on the sociometric status in peer relationship (Figure 3a–c, Table 2). The pooled aggressive behaviors, calculated by summing up the frequency of each behavior, always tended to be higher in the control group compared to the Trp group at the 17th (*p* = 0.060) and 15th (*p* = 0.081) weeks, but the differences disappeared at the 18th week (*p* = 0.138) (Figure 2a). At the 17th week, the Trp exposure-induced alterations were revealed in threat (*p* = 0.087) and kick (*p* = 0.061), while switched to feather peck (*p* = 0.091) at the 18th week (Table 2). In terms of the aggressive tendencies, the within-treatment aggressive behavioral observation at the 19th week revealed a lower frequency in aggressive peck and shorter duration of fight with peck in the Trp group compared to the Saline group (Figure 3d).

### 3.3. Embryonic Trp Exposure Altered Gut Morphology

**Figure 4:**
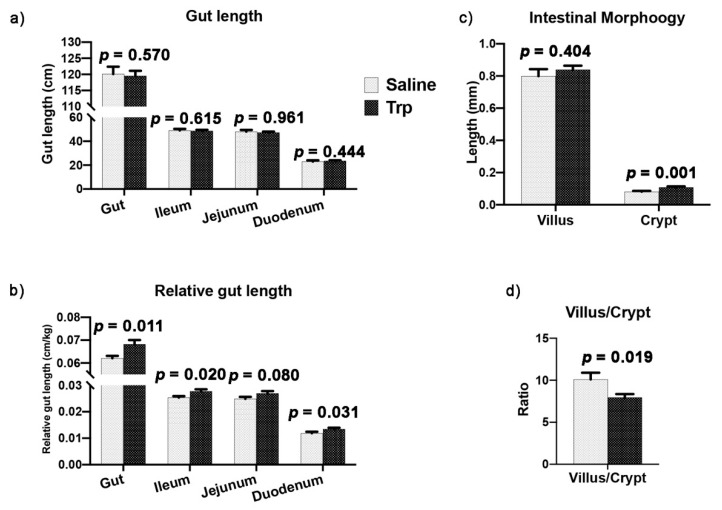
The effect of embryonic Trp exposure on the gut morphology. (**a**–**b**) The absolute and relative length of the entire gut and its sections at the 21st week (n_Saline_ = 11; n_Trp_ = 13). (**c**) The length of villus and the depth of crypt at the ileum-jejunum conjunction at the 21st week (n_Saline_ = 11; n_Trp_ = 13). (**d**) The villus/crypt ratio at the ileum-jejunum conjunction at the 21st week (n_Saline_ = 11; n_Trp_ = 13).

The gut length, including the entire intestine, ileum, jejunum, and duodenum, was similar between the Trp and Saline groups (Figure 4a), while increases in the relative gut length were detected in the Trp group (Figure 4b). Moreover, the morphological analysis revealed that the crypt depth at the ileum-jejunum conjunction was increased by Trp exposure, while the villus/crypt ratio was reduced in the context of similar villus height (Figure 4c,d).

### 3.4. Embryonic Trp Exposure Altered Microbial Profile

**Figure 5:**
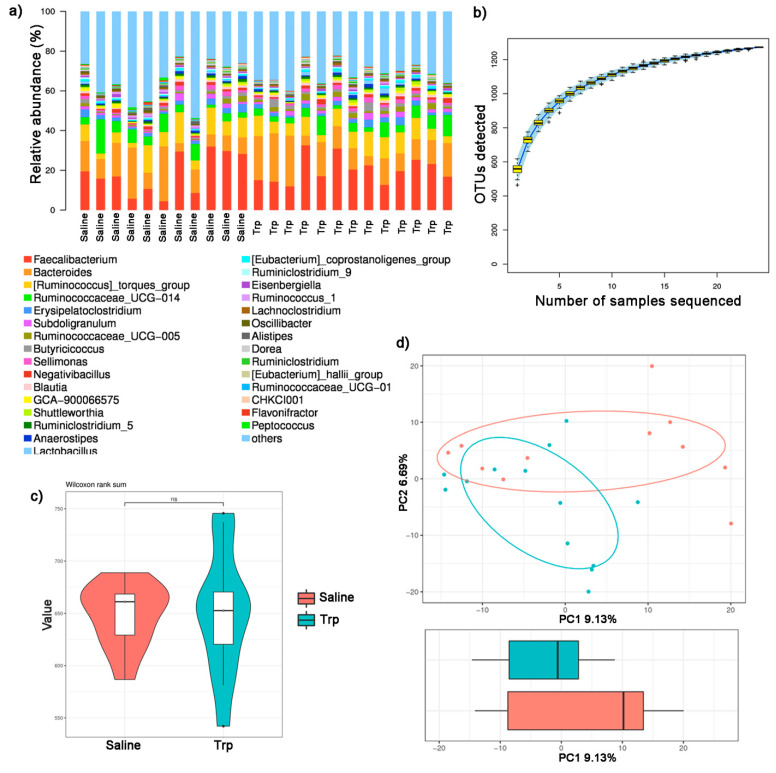
The effect of embryonic Trp exposure on the gut microbial community. (**a**) The relative abundance of bacterial composition in the Trp and Saline groups. (**b**) Species accumulation curve. (**c**) Chao1 alpha-diversity (n_Saline_ = 11; n_Trp_ = 13). (**d**) PCA analysis. ns: no significance.

The raw sequencing data were in FASTQ format and submitted to the NCBI Sequence Read Archive (The BioProject ID: PRJNA762261). A total of 756,365 valid tags from all samples, and 22,041 to 31,567 valid tags were collected from each sample, with an average length of 252.87 to 253.09 bp (Supplementary Table S2). All valid tags were delineated into OTUs (sequence similarity threshold = 97%), resulting in a total of 1272 OTUs, and each region sample contained 465 (Trp5) to 618 (Trp7) OTUs (Supplementary Table S3). The representative tags of each OUT were annotated and defined taxonomy. The gut microbiota community composition of the Trp and Saline groups were further revealed with the top 30 abundant genera listed in each sample (Figure 5a and Supplementary Table S4).

The species accumulation curve rose rapidly at first and then reached saturation with the increase in samples sequenced (Figure 5b), indicating that these samples sequenced could reflect intestinal microbial abundance. No statistical significance was detected in the bacterial community richness, indicated by Chao1 (Figure 5c and Supplementary Table S5), or the mean diversity between the Trp and Saline groups, indicated by Simpson, Shannon and Good’s coverage (Supplementary Table S5). While significant differences in the bacterial composition between the Trp and Saline group were found with PCA (Figure 5d) with a *p*-value of 0.036 revealed by ANOSIM test (Table 3).

**Figure 6:**
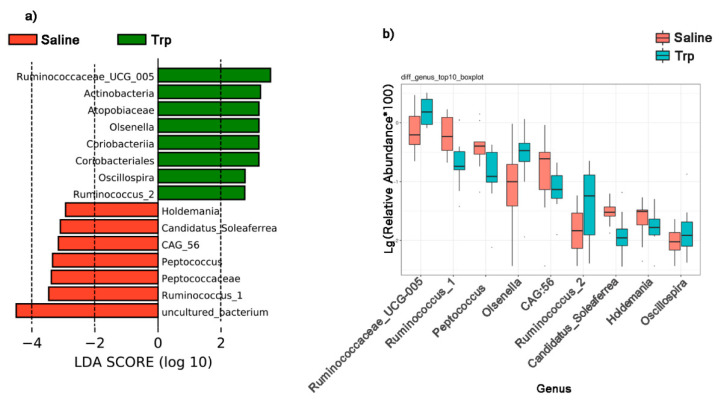
The effect of embryonic Trp exposure on the bacterial taxa abundance. (**a**) The LEfSe analysis reveals the differentially abundant taxa in the Trp and Saline groups by the Kruskal–Wallis test (FDR > 0.05) and with an LDA score of greater than 2.0. (**b**) The Wilcoxon rank sum test reveals the abundance of selected bacterial genus between the Trp and Saline groups (n_Saline_ = 11; n_Trp_ = 13).

The LEfSe analysis revealed the differential abundance in gut microbial communities in the Trp and Saline groups (Figure 6a). The differential species score map revealed four differential taxa (*Olsenella* belonging to the phylum of *Actinobacteria*, and *Ruminococcaceae UCG-005*, *Oscillospira*, and *Ruminococcus_2* belonging to *Firmicutes*) in the Trp group; seven taxa (*Holdemania*, *Peptococcaceae*, *Peptococcus*, *Ruminococcus_1*, *Candidatus_Soleaferrea*, *CAG:56*, and unculture_bacterium belonging to *Firmicutes*) in the Saline group. Moreover, the Wilcoxon Rank Sum Test verified the abundance changes by Trp exposure at the genus level (Figure 6b and Supplementary Table S6). 

**Figure 7:**
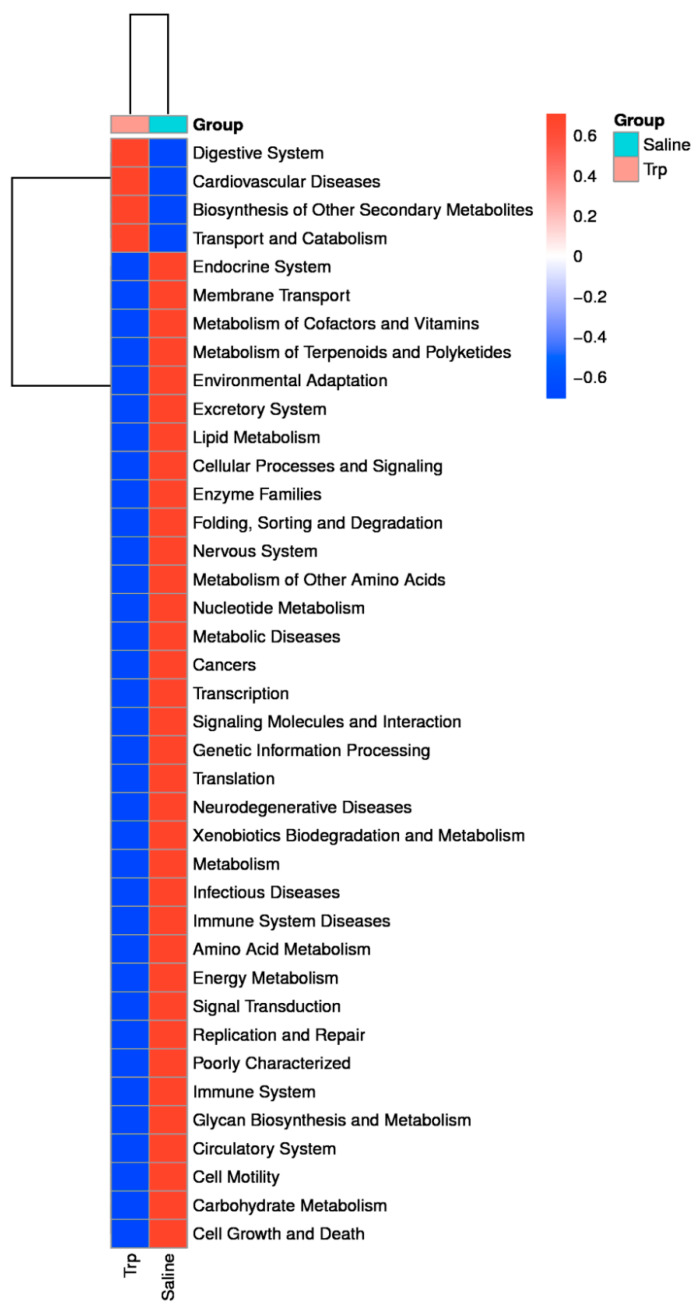
The effect of embryonic Trp exposure on the microbiome function at KEGG L2 level. The heatmap indicates the difference of metabolic pathways between the Trp and Saline groups based on the 16S rRNA gene sequences annotated to KEGG orthologies, which represents the predicted functional composition of the microbiome.

The KEGG analysis revealed the predicted alterations in the gut microbiota functions linked to those of the host’s biological systems (Figure 7): Trp exposure resulted in an enhanced digestive system but weakened endocrine, nervous, and immune systems and related diseases, lipid metabolism, and environmental adaptation, but facilitated the biosynthesis of other secondary metabolites, and transport and catabolism, etc.

### 3.5. Embryonic Trp Exposure Affected Catecholamine and Serotonin Metabolism

**Figure 8:**
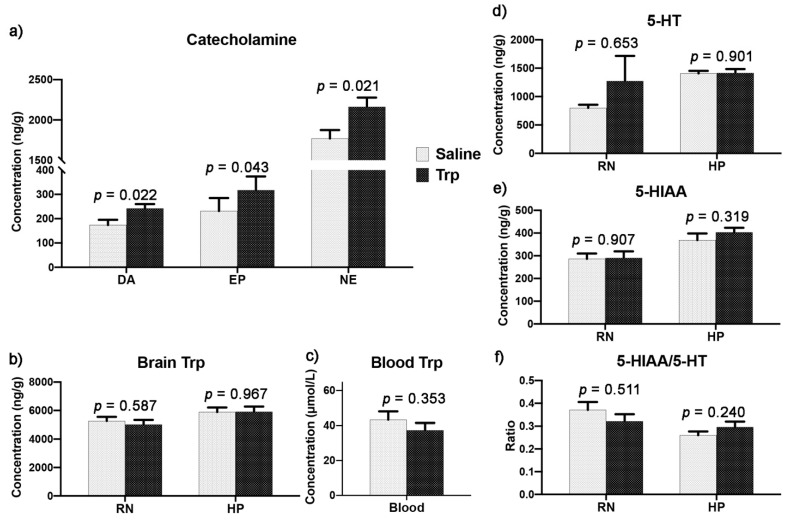
The effect of embryonic Trp exposure on the central 5-HTergic system and hypothalamic CATHs. (**a**). The concentration of DA, EP and NE in the HP. (**b**). The Trp concentration in the RN and HP. (**c**). The Trp concentration in the plasma. (**d**–**e**). The concentration of 5-HT, 5-HT metabolite (5-HIAA) and (**f**). the 5-HT turnover rate in the RN and HP. Data are presented as the concentration of neurochemical per unit wet weight of tissue (ng/g; n_Saline_ = 11, n_Trp_ = 13).

The neurotransmission was detected in the RN and HP. The increases were detected in the hypothalamic catecholamines (CAs) by Trp exposure, including the DA, EP, and NE (Figure 8a). Moreover, the Trp concentrations in the RN and HP as well as in the plasma were not altered (Figure 8b,c). In addition, the 5-HT metabolism, including the 5-HT and 5-HIAA concentrations and the 5-HIAA/5-HT ratio, were similar between the Trp and Saline groups (Figure 8d–f).

## 4. Discussion

Embryonic Trp exposure resulted in lower BW and less aggressiveness during adolescence (7th–19th week of age in chickens), which is consistent with the physical and behavioral characteristics of bullying victimization. Similar to our findings, Trp administration or supplement in the diet has been proven to reduce BW in mice [53], and the excess maternal Trp supplement (5%) has been reported to reduce the fatal and pup BW [54]. Moreover, repeated Trp administrations decrease quarrelsomeness and increase agreeableness, while acute Trp depletion increases aggression in humans [55]. In this study, the Trp exposed birds showed less competitiveness than the Saline birds, especially at early postnatal age. These results may suggest that the prenatal Trp administration reprograms postnatal physiology and behavior. Furthermore, the significance in the aggressive behaviors shifted from threat and kick at the 8th week to feather peck at the 18th week, which is in agreement with the theory. The elevation in the aggressive peck negatively correlates with the nonaggressive pecks, e.g., for playing [55]. It may underline the previous findings that the early life adverse impacts can be buffered and masked by the favorable experience at a later age, such as the environmental enrichment or social support from other birds [56].

Imbalance of gut microbiota is associated with neuroinflammation and psychiatric disorders in humans and various experimental animals [19,20]. Modulation of gut microbiota using prebiotics, probiotics, and synbiotics to regulate the MGB axis has been used as an alternative to antidepressant therapy in humans [56]. In the current study, the reprogrammed MGB axis was found along with the decreased BW and aggression. The low BW in Trp group may be related to the altered gut microenvironment and reduced nutrient absorption surface, indicated by the decreased villus/crypt ratio in the ileum-jejunum junction. Though the alterations in the absolute organ weight and gut length were not detected at the 21st week, the influence of Trp exposure may have existed, as indicated by increased relative gut length at the 21st week, which may have been masked by postnatal environmental influence [57]. Consistently, it has been reported that the difference in the gut length by Trp exposure at E18 was detected at postnatal day 4 and disappeared afterwards [32]. The altered gut microbiota composition reported in this study may be associated with the enhanced digestive system, facilitated nutrient transport and catabolism, and eventually causes the alterations in physiology and behaviors. In addition, gut microbiota functions as a virtual endocrine organ [16], contributing to the gut homeostasis, brain function, and behavioral exhibition through the MGB axis [58]. In the current study, the gut microbiota composition was altered by Trp administration, leading to the treated birds having high abundance of genera *Olsenella* and *Oscillospira* and low abundance of *Holdemania*, with reduced aggressiveness. The high abundance of genus *Olsenella* and family *Ruminococcaceae* and the low abundance of genus *Holdemania* have been reported to be associated with depression [59,60] and ASD [61]. Thus, the alteration in the gut bacterial community may be involved in regulating the psychological activity facing bullying. The abundance in *Olsenella*, on the other hand, is increased in lean people in the Japanese population [62], which works as the only skatole-producing bacterium isolated from pig gut [63] and is positively correlated with the Yale Food Addiction Scale (YFAS) [64]. Moreover, all of the abundance genera in the Trp group had been identified as the core microbes in the crypt of human colon [65]. This is consistent with the increased crypt depth and decreased villus/crypt ratio in Trp exposed roosters, which suggests the coadaptation between the microbiome and gut morphological structure. The increased abundance of the genus *Oscillospira*, the butyrate producer [66] with broad protection against obesity [67], is consistent with the decreased BW in the Trp group. It also hints that the Trp exposed birds with reduced BW and aggressiveness had a modified morphology of the gut and different composition of microbiota.

Maternal stress has been linked to an overactive HPA axis in mammals [68]. Stress-induced overaction of the HPA axis is associated with reactive aggression [69]. Moreover, the HPA axis is intimately involved with many mood disorders, including anxiety, attention deficit hyperactivity disorder (ADHD), depression, etc. [70,71]. In this study, the concentrations of CAs in the HP were increased by embryonic Trp exposure. Striatal DA synthesis is increased in ASD and associated with social defeat [72], who are easier prey for bullies [73]. In addition, DA is the precursor of NE and EP, which contributes to the feelings of pleasure and satisfaction as part of the reward system [74]. Functionally, EP and NE are very similar as neurotransmitters or hormones, both of them work on regulating the fight-or-flight response to stress [75]. The NE reuptake inhibitor, viloxazine, with a second function of inhibiting the activities of the DA receptors and transporters, increases the availability of DA and NE [76] and reduces symptoms of impulsivity and inattention in ADHD, which is directly related to bullying behaviors in males [77]. Moreover, DA and NE reuptake inhibition has been proven to work on regulating the energy homeostasis in both lean and obese mice [78]. The DA-deficient mice are severely hypoactive, adipsic, and aphagic [79], while bilateral hypothalamic DA infusion suppresses feeding in male Zucker rats [80]. Thus, elevated DA in the HP may be underlying the weakened lipid metabolism predicted by KEGG. The NE-deficient mice are slightly hyperphagic [81]. Thus, the Trp exposure may have elevated the concentrations of CAs in the thalamus and hypothalamus to mediate the energy homeostasis and social interaction. In addition, abnormality in the 5-HT metabolism has also been reported in the ADHD, ASD [82] as well as eating disorders [83,84]. However, the difference in the brain 5-HT metabolism or the concentration of Trp in the blood was not detected in this study, which is probably due to the vanishing of maternal influence with age [57]. There results may indicate that the effects of prenatal Trp administration on behaviors are through regulating CAs rather than 5-HT system, though, in contrast, repeating postnatal Trp administration increases 5-HT immunoreactivity in mice [53].

The interaction between gut microbiota and the CNS via the MGB axis is critical in regulating stress coping strategy and ability [16,85]. The HPA axis developmentally cross-talks with gut microbiota [86], which hints the gut microbes’ role in shaping neurogenesis and related behavioral development in offspring. Taking as an example skatole, one of the gut Trp metabolites, produced by *Olsenella* [63], we can see a positive association with the antiatomic connectivity of the amygdala and the functional connectivity of the nucleus accumbens (NAc) [64], by which it is involved in reward learning and addiction [74,87]. The amygdala is constantly scanning our environment to initiate the HPA reaction regulating the fight-flight-freeze-fold response to traumatic events [88,89] as well as to mediate food preference [90]. Moreover, the NAc, a major component of the ventral striatum [91], is related to ingestive behavior [92] and the reward circuit in mood regulation [93]. The NAc releasing DA is necessary to promote the behavioral response to reward-predictive cues [74]. Thus, the metabolic activity of the microbiota may have affected the function and activity of the CNS in feeding and stress coping. In addition, the potential increase in the skatole metabolism following Trp administration may be related to the items predicted by KEGG, including enhanced biosynthesis of some secondary metabolites, facilitated transport and catabolism, and attenuated lipid metabolism as well as weaken environmental adaption. Hence, Trp exposure may have altered the metabolic activity of the microbes and the function of microbiota, affecting the brain function in food intake and mood regulation, by which it further determines the reaction to confront bullying.

This study revealed the relationship between excessive gestational Trp and bullying victimization, i.e., embryonic Trp exposure reduced BW and aggressiveness in roosters. The mechanism may be underlying the reprogrammed MGB axis, i.e., Trp administration causing the differential abundance in gut microbial community which is potentially associated with the increased skatole and butyrate syntheses as well as elevated CA concentrations in the thalamus and hypothalamus. The current results may have revealed one of the mechanisms underlying the maternal stress causing bullying among individuals. It hints to a biological path via modifying the maternal-fetal transfer or early postnatal intervention to avoid or alleviate bullying by improving social adaptation via controlling dietary Trp and medication therapy management during pregnancy.

## Figures and Tables

**Table 1:** Behavioral ethogram.

Behaviors		Description
Aggressive peck		Forceful pecks to the head or neck of the other bird
Body peck	Severe peck	Vigorous pecks to at the feather on the wing, back, or tail, that tend to pull, break or remove the feathers
	Feather peck	Gentle pecks/nibbles at the feathers on the wing, back, or tail
Kick		Forceful extension of leg to make contact with the other bird
Threat		Standing with the neck erected and hackle feathers raised in front of the other bird
Fight with peck		Similar to threat, but at least one aggressive peck is involved
Fight with kick		Similar to threat, but at least one kick is involved

The behavioral ethogram is adjusted based on Dennis et al. (2013).

**Table 2:** The aggressive behavioral observations between BW-paired Trp and Saline birds. Statistical values from PROC GLIMMIX, including sample size (n), F-statistics (F) and probability values (*p*).

	n	F	*p*
Aggressive behaviors (7th week)	7	4.31	0.060
Aggressive peck	7	2.6	0.133
Sever peck	7	2.38	0.149
Feather peck	7	0.15	0.709
Kick	7	4.27	0.061
Threat	7	3.48	0.087
Aggressive behaviors (15th week)	6	3.77	0.081
Aggressive peck	6	1.59	0.236
Sever peck	6	1.38	0.268
Feather peck	6	2.91	0.119
Kick	6	0.66	0.437
Threat	6	0.63	0.444
Aggressive behaviors (18th week)	9	2.43	0.138
Aggressive peck	9	1.38	0.257
Sever peck	9	0.01	0.938
Feather peck	9	3.23	0.091
Kick	9	0.17	0.688
Threat	9	1.95	0.182

**Table 3:** ANOSIM analysis for gut microbiome between the Trp and Saline groups.

Method Name	ANOSIM
Test statistic name	R
Sample size	24
Number of groups	2
Test statistic	0.120826542
*p*-value	0.036
Number of permutations	999

## Data Availability

The raw sequencing data were in FASTQ format and submitted to the NCBI Sequence Read Archive (The BioProject ID: PRJNA762261).

## Supplementary Materials

The supplementary data are available online at https://doi.org/10.5281/zenodo.5938671. Table S1. The exploration of the injection dosages; Table S2. The summary table of sample tag distribution; Table S3. The summary table of sample OTUs abundance and annotations; Table S4. The top 30 abundant genera listed in each sample; Table S5. The estimated OTU richness (Chao1), diversity index (Shannon and Simpson) and estimated sample coverage for all samples; Table S6. The changes in the gut microbiota composition by embryonic Trp exposure at the genus level.
