# Supplementary material for: Embryonic Exposure to Tryptophan Yields Bullying Victimization via Reprogramming the Microbiota-Gut-Brain Axis in a Chicken Model
Source: Nutrients. 2022 Feb 4;14(3):661. doi: 10.3390/nu14030661 (PMC8839409; doi:10.3390/nu14030661)
Supplement: Supplementary file 1 [file nutrients-14-00661-s001.zip › Supplementary Table S1.pdf]

Supplementary Table S1. The exploration of the injection dosages

Exploration	Timing	Dosage	Hatching rate
1st	E0	Intact	0.88
		1mg/ml in 100 µl saline	0.53
		3mg/ml in 100 µl saline	0.40
2nd		Intact	0.88
	E0	50ng in 100 µl saline	0.61
	E12		0.85
3rd	E12	Intact	0.93
		100 µl saline	0.89
		200 µg in 100 µl saline	0.81
		400 µg in 100 µl saline	0.95
		600 µg in 100 µl saline	0.88
		1000 µg in 100 µl saline	1
