# Supplementary material for: Embryonic Exposure to Tryptophan Yields Bullying Victimization via Reprogramming the Microbiota-Gut-Brain Axis in a Chicken Model
Source: Nutrients. 2022 Feb 4;14(3):661. doi: 10.3390/nu14030661 (PMC8839409; doi:10.3390/nu14030661)
Supplement: Supplementary file 1 [file nutrients-14-00661-s001.zip › Supplementary Table S5.pdf]

Supplementary Table S5. The estimated OTU richness (Chao1), diversity index (Shannon and Simpson) and estimated sample coverage for all samples.

| Samples | Simpson | Chao1 | Shannon | Goods_coverage |
|---|---|---|---|---|
| Saline1 | 0.94559521 | 688.832929 | 6.22304128 | 0.99401636 |
| Saline2 | 0.94552455 | 620.10315 | 5.81343038 | 0.99423444 |
| Saline3 | 0.93598124 | 638.353566 | 5.85418 | 0.99405725 |
| Saline4 | 0.94843117 | 586.692883 | 5.73625448 | 0.99500682 |
| Saline5 | 0.96804894 | 668.387596 | 6.29429845 | 0.99386642 |
| Saline6 | 0.95031391 | 668.498192 | 6.16129302 | 0.99408451 |
| Saline7 | 0.90439504 | 616.923557 | 5.62436488 | 0.99421627 |
| Saline8 | 0.95904692 | 671.659062 | 6.07629727 | 0.99395729 |
| Saline9 | 0.89085788 | 641.494566 | 5.5127225 | 0.99370286 |
| Saline10 | 0.90642568 | 661.081262 | 5.83288253 | 0.99380736 |
| Saline11 | 0.91018744 | 665.776215 | 5.68936862 | 0.99347115 |
| Trp1 | 0.94580752 | 664.060649 | 5.90992615 | 0.99401636 |
| Trp2 | 0.94235516 | 627.419703 | 5.75273843 | 0.9942617 |
| Trp3 | 0.92537971 | 581.369233 | 5.30757806 | 0.99477056 |
| Trp4 | 0.88972789 | 652.616001 | 5.70763331 | 0.99401636 |
| Trp5 | 0.94591372 | 542.109639 | 5.69716522 | 0.99526124 |
| Trp6 | 0.89657724 | 703.389084 | 5.70291296 | 0.99353021 |
| Trp7 | 0.94237482 | 737.125396 | 6.17826874 | 0.99311222 |
| Trp8 | 0.94163575 | 670.437593 | 6.16850131 | 0.99419355 |
| Trp9 | 0.9675944 | 745.670073 | 6.5666323 | 0.9929214 |
| Trp10 | 0.95052488 | 649.854741 | 6.24162082 | 0.99408905 |
| Trp11 | 0.92568306 | 620.380445 | 5.86448484 | 0.99413903 |
| Trp12 | 0.92399034 | 663.334843 | 5.66815068 | 0.99339846 |
| Trp13 | 0.94740976 | 596.309036 | 5.73867665 | 0.99423444 |
