# Supplementary material for: Embryonic Exposure to Tryptophan Yields Bullying Victimization via Reprogramming the Microbiota-Gut-Brain Axis in a Chicken Model
Source: Nutrients. 2022 Feb 4;14(3):661. doi: 10.3390/nu14030661 (PMC8839409; doi:10.3390/nu14030661)
Supplement: Supplementary file 1 [file nutrients-14-00661-s001.zip › Supplementary Table S6.pdf]

Supplementary Table S6. The changes in the gut microbiota composition by embryonic Trp exposure at the genus level.

Taxon	Test-Statistic	P	FDR_P	Bonferroni_P	Saline_mean	Trp_mean
Ruminococcus_1	24	0.001998	0.193306	0.257742	0.008165	0.003416
Peptococcus	24	0.002997	0.193306	0.386613	0.005017	0.001943
Ruminococcus_2	31	0.005994	0.257742	0.773226	5.60E-05	0.000534
uncultured_bacterium	32	0.010989	0.279220	1	0.130593	0.067928
Candidatus_Soleaferrea	30	0.011988	0.279220	1	0.000289	0.000159
Ruminococcaceae_UCG-005	31	0.012987	0.279220	1	0.010160	0.017958
Olsenella	34	0.025974	0.478664	1	0.001996	0.004033
Holdemania	36	0.029970	0.483266	1	0.000262	0.000164
Oscillospira	37	0.035964	0.515484	1	5.16E-05	0.000207
CAG-56	37	0.041958	0.541258	1	0.002638	0.000842
